# Supplementary material for: From Overweight to Severe Obesity: Physical Activity and Behavioural Profiles in a Large Clinical Cohort
Source: J Funct Morphol Kinesiol. 2025 Jul 24;10(3):283. doi: 10.3390/jfmk10030283 (PMC12372107; doi:10.3390/jfmk10030283)
Supplement: Supplementary file 1 [file jfmk-10-00283-s001.zip › jfmk-3728158-supplementary.pdf]

Table S1. Age-Adjusted Prevalence of Self-Reported Eating Behaviours by BMI Class.

BMI Class	Skipping Meals (%)	Distracted Eating (%)	Uncontrolled Eating (%)	Snacking (%)	Fast Eating (%)	Night Eating (%)
Overweight	31.2	65.2	76.9	59.6	73.6	14.5
Obesity I	36.7	66.4	80.3	59.1	78.7	18.4
Obesity II	38.0	62.7	83.3	55.3	76.0	16.7
Obesity IIIA	34.3	68.6	94.3	65.7	71.4	25.7
Obesity IIIB	43.8	68.8	100.0	81.2	62.5	18.8
p	0.033*	0.393	0.032*	0.105	0.038*	0.062

The table presents the percentage of participants reporting each eating behaviour, adjusted for age using logistic regression, along with the corresponding p-values for differences across BMI classes.
